# Platelet Activation and the Immune Response to Tuberculosis

**DOI:** 10.3389/fimmu.2021.631696

**Published:** 2021-05-19

**Authors:** Daniela E. Kirwan, Deborah L. W. Chong, Jon S. Friedland

**Affiliations:** Institute for Infection & Immunity, St. George’s, University of London, London, United Kingdom

**Keywords:** tuberculosis, platelets, innate immunity, inflammation, lung fibrosis, anti-platelet drugs

## Abstract

In 2019 10 million people developed symptomatic tuberculosis (TB) disease and 1.2 million died. In active TB the inflammatory response causes tissue destruction, which leads to both acute morbidity and mortality. Tissue destruction in TB is driven by host innate immunity and mediated *via* enzymes, chiefly matrix metalloproteinases (MMPs) which are secreted by leukocytes and stromal cells and degrade the extracellular matrix. Here we review the growing evidence implicating platelets in TB immunopathology. TB patients typically have high platelet counts, which correlate with disease severity, and a hypercoagulable profile. Platelets are present in human TB granulomas and platelet-associated gene transcripts are increased in TB patients versus healthy controls. Platelets most likely drive TB immunopathology through their effect on other immune cells, particularly monocytes, to lead to upregulation of activation markers, increased MMP secretion, and enhanced phagocytosis. Finally, we consider current evidence supporting use of targeted anti-platelet agents in the treatment of TB due to growing interest in developing host-directed therapies to limit tissue damage and improve treatment outcomes. In summary, platelets are implicated in TB disease and contribute to MMP-mediated tissue damage *via* their cellular interactions with other leukocytes, and are potential targets for novel host-directed therapies.

## Introduction

Tuberculosis (TB) is one of the most important infectious diseases of our time. In 2019 10.0 million people developed symptomatic disease and 1.2 million died, with little change in these figures over the past decade (1). Although many patients are never diagnosed or treated, a significant proportion of deaths occurs in individuals who have received appropriate anti-TB treatment (2). This is largely due to the inflammatory immune response to *Mycobacterium tuberculosis* (M.tb), the causative agent of TB. This response is highly complex encompassing both the innate and adaptive immune systems, resulting in severe disease manifestations and facilitating onwards transmission (3). In addition, the co-evolution of M.tb and humans has led to bi-directional adaptations that enable the bacteria to persist in a semi-dormant state, and consequently current treatment regimens require a minimum of six months of therapy to ensure eradication of all viable bacilli within the patient (4). Treatment is further complicated by the rise of single-, multi- and extensively drug-resistant disease.

Moreover, even when successfully treated, patients can suffer long-term impaired lung function and reduced quality of life (5–9). This is long established (10–15) and, given that TB primarily affects adults within the economically active age group, has significant public health and economic implications. Pulmonary rehabilitation leads to improvements in pulmonary function following TB (16), so it is important to identify patients with post-TB sequelae so that they can be appropriately managed pending the development of therapies that can reverse or prevent progression of sequelae including lung fibrosis.

Despite these clear indications that heightened immune activity in TB impacts its morbidity and mortality, current treatment regimens focus solely on bacterial killing. Consequently, there is a drive to develop interventions targeting inflammatory responses in TB. Designed to be given alongside standard treatment, achieving a more controlled inflammatory response could have a number of benefits including improving drug penetration at the site of disease and limiting the damage sustained by lung tissues, and thereby improve both acute and long-term clinical outcomes. This could have a huge impact particularly in the era of rising antimicrobial drug resistance. However, for this to be successful a detailed understanding of the innate response in TB is needed. Accumulating evidence indicates that platelets play an important role and may be key to unlocking therapies designed to improve prognosis.

## Immunopathology in TB: The Matrix Degrading Phenotype

Pulmonary TB is characterized by pathophysiology that is disproportionate to the mycobacterial load, and even in paucibacillary disease, patients may present with severe inflammation. The commonly held belief is that following inhalation mycobacteria are phagocytosed by macrophages, the main innate immune effector cells and hosts for bacterial replication. Macrophages become activated and recruit other innate immune cells including neutrophils (17) which are important early responders to TB infection, involved in both pathogen elimination and in inflammation (18). In addition, a secondary cell-mediated immune reaction involving cross-presentation and priming of CD8+ T cells occurs days after initial antigen exposure (19), known as delayed-type hypersensitivity. These processes can lead to the formation of a granuloma whereby lymphocytes and neutrophils surround infected macrophages to isolate the bacteria; if this containment is unsuccessful, bacterial replication and dissemination occur.

A classic feature of pulmonary TB which is often present at diagnosis is cavitation of the lung. Here, the destruction of lung parenchyma leads to the development of large, air-filled spaces that are relatively immunoprivileged sites where M.tb can proliferate exponentially. The presence of cavities is associated with treatment failure (20) and drug resistance (21), and patients with cavitary disease are highly contagious and represent the main drivers of transmission (22). Cavities are formed through the activity of enzymes called matrix metalloproteinases (MMPs) that are the final effectors of the host innate inflammatory response (23, 24). MMPs are a family of zinc-dependent proteases that collectively degrade all components of the extracellular matrix (ECM), and also have immunological functions including modulating cytokine and chemokine activity, activating defensins, and cleaving proteinase-activated receptors (25, 26). They are tightly regulated by mechanisms such as gene expression, compartmentalization, secretion as proenzymes, enzymatic inactivation, and by specific tissue inhibitors of metalloproteinases (TIMPs). MMPs are responsible for the turnover of normal tissues; if produced in excess they cause tissue damage. In pulmonary TB MMP-1, interstitial collagenase, is the main effector of lung degradation and cavity formation (24, 27). MMP concentrations in plasma and bronchoalveolar lavage fluid (BALF) have consistently been shown to be elevated in patients with acute TB, and correlate with clinical and radiological disease severity (27–30). Genetic polymorphisms that result in greater MMP-1 secretion in pulmonary TB have been identified as a risk factor for developing fibrosis following TB (31).

Persistent inflammation in TB can lead to fibrosis, and following TB treatment many patients suffer long-term lung damage, with reported rates of impairment of up to 68% (8, 32–34). Affected patients can have restrictive, obstructive, or mixed patterns on spirometric testing (34) and this can manifest as various chronic lung diseases including pulmonary fibrosis, emphysema, and bronchiectasis (35). Consequently, TB survivors have shortened life expectancies, with one model predicting an average loss of 3.6 years of life (36), and this is associated with a reduced perceived mental health-related quality of life (HRQoL) even after microbiological cure (37). The extent of radiological disease severity at diagnosis is the most important determinant of residual pulmonary function post treatment (33, 38, 39), however the mechanisms that link initial bacterial infection to the development of fibrosis are poorly understood.

## Clinical Evidence for the Role of Platelets in the Response to TB

The initial detection of M.tb and subsequent rapid innate immune response may be initiated and accelerated by platelets. Numerous observational studies report thrombocytosis in patients with TB (40–45) and, in some, platelet count correlated with levels of acute phase reactants such as C-reactive Protein (CRP) and with disease severity (45–47). Sahin et al. identified thrombocytosis in 44/100 pulmonary TB patients, and they had higher plateletcrit and platelet distribution width (PDW) than healthy controls. Thrombocytosis was associated with clinically and radiologically advanced disease, and platelet count correlated with CRP and erythrocyte sedimentation rate (ESR) values in pulmonary TB patients but not in patients with community acquired pneumonia (48). In a South Korean study, platelet counts were higher in TB patients than in controls, and in TB patients, the mean platelet volume (MPV) correlated with CRP levels (49). Other studies, however, have associated thrombocytopenia with tuberculosis. For example, in 128 Indian patients, thrombocytopenia was observed in 37.5% (50).

Platelet-associated gene transcripts are upregulated in TB patients (51). As platelet RNA is formed in megakaryocytes prior to platelet formation, the megakaryocyte itself may be influenced by disease states and platelets may be pre-programmed to respond specifically to TB. This has been shown in other diseases such as cancer, where the platelet transcriptome can be used to accurately diagnose various cancers and may predict outcomes (52).

Changes in platelet structure and function have been described in TB patients. Patients with acute TB have increased numbers of alpha granules (53) which contain pro-inflammatory mediators such as tumor necrosis factor alpha (TNFα) and interleukin-1 beta (IL-1β), known to be elevated in TB patients (54). In contrast, patients with chronic TB exhibited elongated platelets and increased numbers of alpha and dense granules. The latter contain a range of mediators including adenosine diphosphate (ADP), adenosine triphosphate (ATP), serotonin, and ionized calcium which is involved in the coagulation cascade. These differences may reflect distinct functional roles at different disease stages, with a predominantly pro-inflammatory phenotype in acute TB and a mixed inflammatory-thrombotic state in chronic disease.

Increased platelet activity can be detected by quantifying markers released from platelet granules upon activation. Elevated plasma concentrations of platelet factor 4 (PF4; CXCL4), a component of alpha granules specific to platelets, are found in patients with pulmonary TB and correlate with radiological disease severity (55). In a cohort of Peruvian patients with newly diagnosed, drug-sensitive smear-positive pulmonary TB, plasma levels of PF4, platelet-derived growth factor (PDGF)-BB, C-C motif chemokine ligand 5 (CCL5; RANTES), MMP-9, soluble CD40 ligand (sCD40L), and Pentraxin-3 (PTX-3; TNF-stimulated gene (TSG)-14) were elevated at baseline compared to age- and sex-matched controls. Fifty percent of these patients were followed up during their anti-TB treatment, and the plasma concentrations of all of these markers increased at Day 14 and then decreased, returning to normal by Day 60 (56). Levels of platelet-derived mediators such as PF4 have been shown to correlate with disease progression and severity in other chronic inflammatory conditions including inflammatory bowel disease (57), atherosclerosis (58), and rheumatoid arthritis (59); therefore, platelet-derived mediators may also drive inflammatory processes in TB, and could be important in the resolution of inflammation and/or development of fibrosis.

## Pre-Clinical Evidence for the Role of Platelets in the Response to TB

Platelets are present at the site of TB disease either as a result of extravasation of platelets that localize to the lesion, or secondary to platelet biogenesis within the lung itself. Megakaryocytes have been identified within the lungs and may have the capacity to scale up platelet generation in response to specific stimuli (60). This would support a role of platelets as first responders in M.tb infection, placing them at close proximity to any mycobacterial intruders. Lung tissue from M.tb-infected Balb/C mice has demonstrated the presence of the platelet marker CD41 which is not detected in uninfected lung. CD41 expression mostly occurred in association with anucleate cells consistent with the morphology of platelets, predominantly within the alveoli, but also in nucleated cells, indicating platelet phagocytosis and/or adherence to leukocytes (56). Similarly, platelet-specific marker CD42b was observed within epithelioid cells and multinuclear giant cells in human TB lung granulomas (61). Microthrombi occur around TB cavities and have been proposed to prevent dissemination (62). Platelet aggregations and platelet-neutrophil adhesions have been observed within pulmonary lesions and mycobacteria have been visualized within platelets, located mainly alongside the mitochondria (53). Such leukocyte-platelet interactions may be critical in regulating the immune response in TB. PDGF-BB, P-selectin, and RANTES concentrations were elevated in BALF from TB patients compared to patients with non-TB respiratory disease. The formation of platelet-leukocyte aggregates also correlates with secreted levels of platelet-derived mediators in other inflammatory diseases such as atherosclerosis (63). To further support the notion that platelet activity may be involved in immune responses in TB, concentrations of P-selectin, a platelet activation marker, positively correlate with levels of well-characterised markers of disease severity including IL-1β, MMP-1, -3, -7, -8 and -9 in TB patients (56).

## Platelet-Leukocyte Signaling and Cellular Interactions in TB

Platelets can sense pathogens directly *via* expressed Toll-like receptors (TLRs) such as TLR2 and TLR4 (64, 65) to lead to activation and release of reactive oxygen species (66) and pro-inflammatory cytokines (67). Activated platelets also interact directly with leukocytes to facilitate cellular recruitment towards the site of infection (68). These adhesive interactions can form platelet-monocyte aggregates (PMA) and platelet-neutrophil aggregates (PNA) which lead to cell activation and enhance immune function such as cytokine or MMP production. Platelets can stimulate neutrophils to release neutrophil extracellular traps (NETs) (69), which are increased in the plasma of TB patients (70) and have been associated with severe TB-associated lung damage and subsequent sequelae (71). These adhesive interactions are mediated by the two main families of cell adhesion molecules, selectins and integrins.

Selectins are expressed on most leukocytes and consist of three family members, L-, P- and E-selectin (CD62L, CD62P and CD62E respectively). These single transmembrane glycoproteins initiate the leukocyte adhesion cascade to aid leukocyte tethering and rolling along inflamed endothelium prior to transmigration (72). Platelets only express P-selectin, which is translocated from alpha granules to the surface membrane in response to agonists such as thrombin, and can be measured in soluble form as a marker of platelet activation; soluble P-selectin levels are higher in TB patients compared to healthy controls (56, 73). P-selectin ligation can activate platelets leading to aggregation, enhanced adhesion, and activation of other platelet expressed integrins such as GPIIb/IIIa (74). Moreover, P-selectin on platelets can interact with P-selectin glycoprotein ligand-1 (PSGL1) expressed on leukocytes to form PMA (75). This interaction has a central role in the pathology of chronic inflammatory diseases including atherosclerosis (76) and heart disease (77). Increased circulating PMA are found in TB patients compared to healthy controls (78), and treatment of M.tb-infected whole blood with anti-P-selectin antibody decreases PMA (78).

Integrins are composed of two subunits, α and β, to form heterodimers that facilitate platelet binding to inflamed endothelium or leukocytes. Platelets express three families of integrins, β1 (CD29), β2 (CD18), and β3 (CD61). β1 integrins recognize sub-endothelial ECM proteins such as collagen (79) or endothelial ligands like VCAM-1 (CD106) (80), whilst β2 integrins bind to intracellular cell adhesion molecule-1 or -2 (ICAM-1/CD54 or ICAM-2/CD102), found on the endothelium, epithelium, or platelets (81, 82). Platelets express two β3 integrins, αvβ3 and αIIbβ3 (GPIIb/IIIa or CD41/CD61). αIIbβ3 is the most dominant integrin expressed on platelets (83) and can recognize arginine-glycine-aspartic acid (RGD)-containing ligands such as fibrinogen and fibrin (84). Ligation of integrins on platelets initiates “inside-out” or “outside-in” signaling pathways to result in functional outcomes such as cell activation, adhesion, or degranulation (85).

Other important adhesive interactions demonstrated between platelets and leukocytes include the binding of surface expressed glycoprotein-Ibα (GPIbα, CD42a), part of a receptor complex that binds von Willibrand factor (vWF), to αMβ2 (CD11b/CD18, Mac-1) on monocytes (86). In addition, platelets express ICAM-2 (87), the ligand for αMβ2 and αLβ2 (CD11a/CD18, LFA-1) expressed on monocytes. In atherosclerosis platelet-derived CD40L (CD154) can interact with CD40 (TNF receptor) expressed on leukocytes to form platelet-leukocyte aggregates (88). Whilst these cellular interactions have been observed *in vitro* or in animal models, the specific interactions important in PMA formation in TB patients remain unclear and require further investigation.

In TB, cellular networks involving resident and influxing leukocytes, stromal cells, and other cells enable amplification of initial responses, leading to secretion of MMPs and other pro-inflammatory mediators (89). There is evidence that cross-talk between platelets and leukocytes, particularly monocytes, may be key to driving TB immunopathology. Platelets have been shown to significantly upregulate the MMP secretion from M.tb-infected monocytes (56) and are important in the development of granulomas and macrophage differentiation in TB (61). M.tb-infected monocytes co-cultured with platelets may phagocytose them and differentiate into larger, multinucleated giant cells with enhanced phagocytosis and shared traits with multinucleated giant cells observed in TB granulomas. Moreover, on extended culture, macrophages differentiated with both platelets and M.tb upregulate genes involved in leukocyte chemotaxis, including CXCL5 and PPBP/CXCL7, and ECM receptor interactions compared to those cultured with M.tb alone. Although the effect of platelets on inflammatory responses in TB depends upon the stage, site, and severity of TB infection, they appear to predominantly steer monocyte differentiation towards an anti-inflammatory phenotype: the presence of M.tb does not affect cytokine secretion from platelets themselves, but has been shown to raise IL-10 and reduce TNFα secretion by platelet-transformed macrophages (61). Similarly, M.tb-infected peripheral blood mononuclear cells (PBMCs) incubated with platelets led to decreased secretion of pro-inflammatory cytokines TNFα, IL-1β, IL-6 and IFNγ but increased IL-10 compared to M.tb-infected PBMCs incubated without platelets (78).

What remain unexplored are the mechanisms by which platelets induce leukocyte activation during M.tb infection to cause reported cellular responses. Data from *in vitro* studies utilizing uninfected cells show that following engagement of platelet expressed P-selectin and PSGL1 on leukocytes, signal transduction pathways are initiated to enhance tyrosine phosphorylation and activation of mitogen-activated protein kinase (MAPK) (90). MAPK are phosphorylation-dependent signal-transducing enzymes involved in immune responses and cellular regulation (91). The p38 and extracellular signal-related kinase (ERK)/MAPK pathways are key in regulating MMP and cytokine secretion during M.tb infection (92–96). Therefore, we hypothesize that P-selectin-PSGL1 binding between platelets and leukocytes in M.tb infection could lead to MMP and pro-inflammatory cytokine gene and protein production *via* MAPK signaling, although this requires confirmation. In contrast, phosphatidyl inositol 3-kinase (PI3K) signaling negatively regulates MMP-1 secretion from M.tb-infected macrophages (97). PSGL1 activation can induce signaling *via* PI3K (98) and activation of mTOR and Rho-associated kinases (ROCKs) in macrophages to facilitate cell motility and phagocytosis (99). Whether platelets interacting with macrophages during M.tb infection also utilize PSGL1-PI3K/mTOR signaling is unknown.

**Figure 1:**
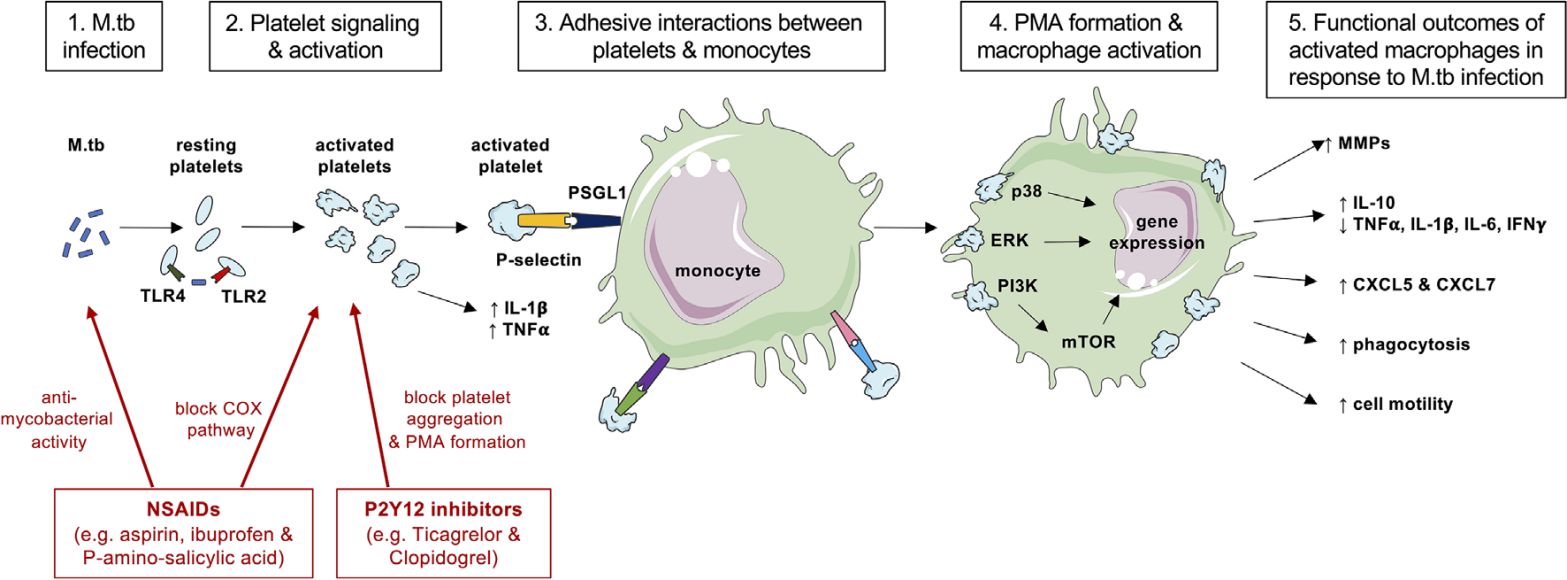
A summary of cellular interactions between platelets and monocyte during M.tb infection and actions of anti-platelet therapies. Platelets may be able to directly sense M.tb infection (step 1) *via* TLR2 and TLR4 to become activated and degranulate to release pro-inflammatory cytokines (step 2). Activated platelets upregulate surface P-selectin expression and bind to PSGL1 on monocytes (step 3) leading to PMA formation (step 4). Ligation of PSGL1 can initiate signal transduction *via* MAPK and induce gene expression (step 4) to result in cellular functional outcomes such as MMP and cytokine production or phagocytosis (step 5). Anti-platelet agents such as non-steroidal anti-inflammatory drugs (NSAIDs) and P2Y12 inhibitors (shown in red) may exhibit direct anti-mycobacterial activity on M.tb or block the actions of platelets to decrease PMA formation and MMP expression that cause collateral host tissue damage. This figure was created with images adapted from Servier Medical Art by Servier. Original images are licensed under a Creative Commons Attribution 3.0 Unported License.

Taken together, these studies indicate that platelets have a key role in directing cellular outcomes in response to M.tb through interactions with cell adhesion molecules and by maneuvering intracellular signaling pathways as summarized by **Figure 1**. A better understanding of these cellular interactions may reveal which signaling mechanisms could be targeted to decrease TB immunopathology.

## Anti-Platelet Agents for the Treatment of TB

Imbalances in eicosanoids have been associated with the development of TB (100, 101). Eicosanoids are lipid mediators derived from the activity of the enzymes cyclooxygenase (COX)-1 and -2 on arachidonic acid, which enter either the cyclooxygenase or the lipoxygenase pathway. The cyclooxygenase pathway leads to prostaglandins D, E and F, prostacyclin, and thromboxane A2 (TXA A2) production. Prostaglandins are powerful vasodilators, inhibit platelet aggregation, and act as signaling molecules linking the innate immune system to acute inflammatory pathways (102), whereas thromboxanes are vasoconstrictors that trigger platelet aggregation (103). The lipoxygenase pathway causes the production of lipoxins, which reduce pro-inflammatory cytokine production and neutrophil recruitment (104). Non-steroidal anti-inflammatory drugs (NSAIDs) inhibit COX-1 and -2, and include aspirin (Acetylsalicylic acid, ASA), the most commonly prescribed anti-platelet agent. Aspirin irreversibly inhibits both COX enzymes and displays powerful anti-thrombotic effects at low doses as well as anti-inflammatory activity at higher doses.

Repurposing anti-platelet and anti-inflammatory NSAIDs for use in TB is attractive because they are already approved for use and have well-established safety profiles. Data indicate they may be effective at enhancing TB control (105); animal and human studies are presented in **Table 1**. NSAIDs have some direct anti-mycobacterial activity (104, 109, 113, 116, 124, 125), directly interfering with M.tb metabolism *via* downregulation of genes involved in energy production (126). However, their main effect in TB is likely to be due to their action on the host immune system, particularly by modulating inflammatory pathways that are active in TB disease (104, 107, 111, 116, 117). Administration of ibuprofen to TB-infected C3HeB/FeJ mice led to fewer and smaller lung lesions, decreased neutrophil infiltration, reduced bacillary load, and enhanced survival (115). The effects of NSAID treatment may be either protective, by enabling effective clearance of pathogens, or harmful by causing tissue destruction, particularly where immune activity is disproportionate to the magnitude of the infective insult (118). This will depend upon multiple factors including timing, route of administration, and dose, all of which must be considered when proposing NSAIDs as a potential adjunctive treatment.

**Table 1:** Animal and human studies investigating the effects of anti-platelet agents in tuberculosis.

Reference	Anti-platelet agent(s) tested	Host species/patients studied	Intervention	Summary of findings
**Animal studies**				
Shroff *et al*, 1990 (106)	Indomethacin	Swiss white mice	Six intraperitoneal injections of indomethacin, 50µg per mouse, given at 12 hour intervals; immunization with intraperitoneal *M. vaccae* 12 hours after the last dose	Indomethacin-treated mice showed a delayed type hypersensitivity response, whereas non-treated mice did not.
Hernández-Pando *et al*, 1995 (107)	Indomethacin	Male 6-8 week old BALB/c mice immunized with culture filtrate proteins (CFP) from H37Rv M.tb delivered *via* endotracheal route	20mg/kg cyclophosphamide or 5mg/kg indomethacin given by intraperitoneal injection one day prior to endotracheal challenge with CFP	Induction of granulomas with sepharose beads coated with CFP reduced antibody production and delayed-type hypersensitivity responses to mycobacterial antigens; this was reversed with the administration of cyclophosphamide and indomethacin.
Rangel Moreno *et al*, 2002 (108)	Niflumic acid	Male 6-8 week old BALB/c mice infected with H37Rv M.tb *via* endotracheal route	500µg/ml niflumic acid given by intragastric cannula twice per day	Mice treated with niflumic acid early in infection produced greater expression of TNF-α, IL-1α, and IFN-γ, with a reduction in inhaled nitric oxide synthase (iNOS) expression and an increased bacterial load. Mice treated after 60 days of infection showed increased pro-inflammatory cytokine concentrations, a striking increment of iNOS expression, and reduced bacillary load.
Dutta *et al*, 2004 (109)	Diclofenac sodium	45 mycobacterial strains including drug-sensitive and -resistant strains in Kirchner’s liquid culture medium; male Swiss Albino mice infected *via* intraperitoneal route with M.tb H37Rv102	Mycobacteria were tested with increasing concentrations of diclofenac sodium. Infected mice were injected with 10mg/kg diclofenac sodium daily for 6 weeks	Diclofenac sodium exhibited bactericidal activity in most mycobacterial strains at a range of 15-25µg/ml. Diclofenac sodium treatment of infected mice resulted in reduced macroscopic lesions and a reduction in mycobacterial load.
Hernández-Pando *et al*, 2006 (110)	Niflumic acid (a COX-2 inhibitor) and soluble betaglycan (an anti-TGF-β agent)	Male 6-8 week old BALB/c mice infected with H37Rv M.tb	30µg soluble betaglycan administered twice a week by intraperitoneal route from 30 days post infection, with/without 500µg niflumic acid administered twice a day by intragastric cannulation, or pan-specific TGF-β antibodies. Mice euthanased at 15, 30, 45, and 60 days	Compared to control animals, treatment produced higher expression of TNF‐α, IFN‐γ, IL‐2, iNOS and lower expression of IL‐4, with decreased lung fibrosis and bacillary load; there was a higher inflammatory response manifested as a greater area of lung affected by pneumonia. The addition of niflumic acid was synergistic, with reduced bacillary load and increased TNF‐α.
Byrne *et al*, 2007 (111)	Aspirin and ibuprofen	BALB/c mice infected with M.tb H37Rv by aerosol administration	Oral treatment of aspirin (10, 20, 40 mg/kg) and ibuprofen (10, 20, 40 mg/kg) +/- pyrazinamide (150 mg/kg) daily for 1 month	Aspirin and ibuprofen had no effect alone, but increased bactericidal activity of pyrazinamide when given in combination.
Byrne *et al*, 2007 (112)	Aspirin and ibuprofen	Four week old BALB/c mice infected with M.tb H37Rv by aerosol administration	Aspirin or ibuprofen (10-40mg/kg) with or without isoniazid (25mg/kg) administered by oral gavage five times per week for one month. Mice were euthanized one day after treatment completion	Co-administration of aspirin and isoniazid antagonized the antimycobacterial activity of isoniazid. Ibuprofen had no interaction with isoniazid.
Dutta *et al*, 2007 (113)	Diclofenac	Male Swiss Albino mice infected *via* intraperitoneal route with M.tb H37Rv	Infected mice treated with diclofenac 10µg/g or streptomycin 150µg/g, alone or in combination, daily for 4 weeks	Diclofenac and streptomycin both resulted in improved survival, reduced bacillary count, and reduced splenic weight. The two drugs acted synergistically
Peres-Buzalaf *et al*, 2011 (114)	Celecoxib and MK-886 (5-LO activation protein inhibitor)	Male 5-8 week old BALB/c mice infected intratracheally with M.tb H37Rv strain	Oral treatment with celecoxib 5mg/kg/0.5ml, and/or MK-886 5mg/kg/0.5ml, 1h prior to infection with M.tb and again every 24h for 60 days	Celecoxib enhanced 60-day survival from 86% to 100% and reduced lung bacterial burden; conversely, MK-886 reduced 60-day survival from 86% to 43% and increased lung bacterial load.
Vilaplana *et al*, 2013 (115)	Ibuprofen	C3HeB/FeJ pathogen-free mice infected intravenously with M.tb H37Rv	Ibuprofen 80mg/kg given orally 3 or 4 weeks post infection.	Ibuprofen treated animals had a reduction in size and number of lung lesions, bacillary load, and improved survival.
Marzo *et al*, 2014 (116)	Aspirin and ibuprofen	C3HeB/FeJ and C3H/HeN pathogen-free mice infected intravenously with M.tb H37Rv	Aspirin 3mg/kg, sodium heparin 20 UI/kg, ibuprofen 80mg/kg orally	Aspirin administration reduced bacterial load, attenuated the severity of histopathological changes, and improved survival. Aspirin and ibuprofen-treated C3HeB/FeJ and C3H/HeN mice had lower levels of pro-inflammatory mediators including TNF-a, IL-6, IL-17 and CXCL5 than untreated C3HeB/FeJ mice.
Kroesen *et al*, 2018 (104)	Aspirin	C3HeB/FeJ infected with M.tb H37Rv Pasteur strain	Aspirin 3mg/kg given alone or in combination with anti-TB treatment	Low-dose aspirin reduced bacterial load and increased survival, reduced lung pathology, decreased production of pro-inflammatory cytokines and delayed neutrophil recruitment and T cell responses. In combination with anti-TB treatment aspirin enhanced survival and reduced lung pathology.
Hortle *et al*, 2019 (117)	Aspirin, and tirofiban and eptifibatide (specific inhibitors of glycoprotein IIb/IIIa–fibrinogen binding)	Zebrafish infected with *M. marinum*	Infected zebrafish treated with 10µg/ml aspirin, 20µg/ml tirofiban, 10uM eptifibatide, or 5uM warfarin, or control	Mycobacterial burden correlated with thrombocyte density. Aspirin, tirofiban, and eptifibatide reduced mycobacterial burden by inhibiting thrombocyte-granuloma interactions.
Mortensen *et al*, 2019 (118)	Celecoxib and ibuprofen	CB6F1 mice infected with M.tb Erdman strain *via* aerosol or intravenous (IV) routes	Celecoxib or ibuprofen administered orally in mouse feed 4-12 weeks post infection	Aerosol-infected mice treated with celecoxib or ibuprofen had higher bacillary burdens in lung and spleen and there was no difference in pulmonary immune cell infiltration or cytokine levels measured in plasma or lung homogenates. Celecoxib led to increased bacterial burden and altered function/differentiation of Type 1 helper T cells in mice re-infected following antibiotic treatment. IV-infected mice had reduced inflammation and bacterial burden following ibuprofen treatment.
**Human Studies**				
Misra *et al*, 2010 (119)	Aspirin	Patients with TB meningitis diagnosed based on clinical, magnetic resonance imaging (MRI), and cerebrospinal fluid (CSF) criteria	118 patients randomized to receive 150mg aspirin per day or placebo. All patients received standard treatment with rifampicin, isoniazid, pyrazinamide and ethambutol with/without corticosteroids	Aspirin resulted in insignificantly fewer strokes (24.2% versus 43.3%, OR 0.42, 95%CI 0.121-1.39) and significantly reduced mortality (21.7% versus 43.4%, p=0.02) compared to placebo. Aspirin was well tolerated.
Schoeman *et al*, 2011 (120)	Aspirin	Children with TB meningitis	146 children randomized to placebo, low-dose aspirin (75mg/day), and high-dose (100mg/kg/day) aspirin, alongside standard treatment plus prednisolone for the first month of treatment	There was no benefit in morbidity or mortality.
Mai *et al*, 2018 (121)	Aspirin	HIV-uninfected adults with TB meningitis	120 patients randomized to aspirin (81mg/day or 1000mg/day) or placebo added to the first 60 days of anti-tuberculosis drugs plus dexamethasone	There was no difference in rates of gastro-intestinal or cerebral bleeding, new brain infarction, or death by 60 days. There was a reduction in new infarcts and deaths by day 60 in aspirin-treated participants with microbiologically confirmed TBM compared to placebo.
Lee *et al*, 2019 (122)	Antiplatelet agents including aspirin	Retrospective cohort study of incident TB cases in the Taiwan National Tuberculosis Registry between 2008 and 2014	9,497 antiplatelet users (including 7,764 aspirin users) compared to 8,864 non-users	After 1:1 propensity score matching, antiplatelet use was associated with longer survival (adjusted hazard ratio (HR): 0.91, 95% confidence interval (CI): 0.88-0.95, p < 0.0001) but no increase in major bleeding risk (p=0.604).
Wang *et al*, 2020 (123)	Aspirin	Patients with pulmonary TB and type 2 diabetes mellitus	348 patients randomized to aspirin (100mg/day) or placebo. 168 patients completed the trial and were included in analysis	Erythrocyte sedimentation rate and C-reactive protein levels were lower (p=0.000), the sputum-negative conversion rate was higher (86.7% versus 53.8%, p=0.031), and the number and size of cavities was smaller in the aspirin-treated than the placebo-treated group.

Data from TB patients suggest that anti-platelet agents can be beneficial. One of the first drugs in use for TB, P-amino-salicylic acid (PAS), is an aspirin homologue that was deemed effective despite an unknown mechanism of action when it was first given to a patient in 1944. We showed that PAS modulates anti-inflammatory immune activity, and suppresses MMP-1 secretion through a PGE2-dependent mechanism without affecting M.tb replication (93). PAS therefore represents the first example of a host-directed therapy used to treat TB. More recently, a Taiwanese population-based study of patients with drug-sensitive TB found that use of antiplatelet drugs was associated with significantly improved overall survival and a lower 12-month mortality rate, as well as lower rates of smear positivity and fewer cavities. Benefits were greater with aspirin compared to non-aspirin anti-platelet agents such as clopidogrel (122). In a randomized controlled trial, giving 100mg aspirin to diabetic patients with pulmonary TB resulted in decreased secretion of inflammatory mediators including ESR and CRP, a higher sputum-negative conversion rate (87% versus 54%), and fewer and smaller cavities following treatment, compared to placebo-treated control patients (123). In TB meningitis, both inflammation and thrombosis are associated with higher mortality and complications including stroke in survivors; randomized controlled trials of aspirin have shown benefits in morbidity and mortality (119, 121). Further studies are currently underway to evaluate NSAIDS including meloxicam and ibuprofen as adjuncts to standard TB treatment regimens (127–129).

Drugs blocking other platelet signaling pathways are potentially important in the management of TB. ADP is the endogenous ligand for P2Y12 and P2Y13 receptors, which are implicated in platelet aggregation. ADP induces MCP-1 expression to enhance macrophage migration, and P2Y13 expression is increased in TB patients (130). Ticagrelor and clopidogrel, both P2Y12 inhibitors, given to healthy volunteers significantly reduced inflammatory and prothrombotic mechanisms including PMA formation and pro-inflammatory cytokine release following endotoxin challenge (131). In a double-blind placebo-controlled study a single dose of oral ticagrelor reduced PMA formation in healthy volunteers, and this was associated with an increase in pro-inflammatory cytokines in blood exposed to the TLR2 ligand Pam3CSK4 and a decrease in blood exposed to TLR4 ligand LPS, suggesting that platelets may differentially modulate cytokine responses depending upon the receptors involved (132). The role of such compounds and of inhibitors of platelet signaling pathways, proposed in **Figure 1**, as potential therapy targets in TB, are not yet known but merit detailed investigation and this represents a major avenue of future research.

## Conclusions and Future Directions

In this review, we have presented substantial evidence that platelets are a key component of the innate immune response to tuberculosis. Both observational and experimental data show that the administration of anti-platelet agents in patients with TB may be effective at limiting disease manifestations and improving long-term outcomes in patients who are successfully treated. These findings are highly promising, however much remains to be understood about the exact mechanisms of platelet engagement with mycobacteria and other cells in the orchestration of the complex immune response to M.tb. Research is needed to ascertain the effect of therapeutic blockade of specific targets within the platelet on downstream signaling and leukocyte activity. In particular, the timing of anti-platelet administration will be of utmost importance to ensure optimal use, enabling interruption of harmful tissue destructive processes whilst preserving, or even enhancing, the immune system’s anti-infective properties.

## Author Contributions

All authors contributed to the article and approved the submitted version.

## Funding

DK is supported by MRC Clinical Research Training Fellowship (MR/P019978/2).

## Conflict of Interest

The authors declare that the research was conducted in the absence of any commercial or financial relationships that could be construed as a potential conflict of interest.
